# Core Microbial Taxa Strengthen Root Microbial Network Stability Under Drought Stress

**DOI:** 10.1111/1462-2920.70307

**Published:** 2026-04-29

**Authors:** Keren Wu, Hang‐Wei Hu, Dorin Gupta, Yuan Li, Zi‐Yang He, Feng Wang, Ji‐Zheng He

**Affiliations:** ^1^ School of Agriculture, Food and Ecosystem Sciences, Faculty of Science The University of Melbourne Parkville Victoria Australia; ^2^ ARC Research Hub for Smart Fertilisers The University of Melbourne Parkville Victoria Australia; ^3^ Grasslands and Sustainable Farming, Production Systems Unit Natural Resources Institute Finland Kuopio Finland

**Keywords:** core taxa, drought stress, growth stages, microbial network stability, rhizosphere soil and root

## Abstract

Drought stress is intensifying globally, but its effects on plant‐associated microbiome diversity and stability remain poorly understood. We grew wheat under drought stress and sampled bulk soils, rhizosphere soils and roots across three growth stages to quantify microbial diversity, co‐occurrence network stability and the contributions of core taxa to network stability. Drought affected microbial diversity depending on microbial kingdoms, plant niches and growth stages. We further found that drought stress reduced the complexity and stability of microbial networks in the rhizosphere soils while enhancing those in the roots, mainly through shifts in the abundances of core taxa (i.e., taxa that are widely distributed across samples, specific to drought stress and highly connected in the network). Three types of analyses (shared operational taxonomic units, network keystone nodes and taxa with high specificity and occupancy values) were employed to identify the core taxa enriched in the roots under drought stress, including *Glycomyces* and *Thermoactinomycetaceae*, which were typical drought‐tolerant taxa that are important for maintaining root microbial network stability. Environment stress usually disrupts microbial community stability, but we found drought stress enriched core taxa, enhancing drought‐tolerant crop root microbiomes stability. Our findings provide a blueprint for enhancing crop stress tolerance via microbiome manipulation.

## Graphical abstract

This study evaluates different drought stresses on microbial diversity and network stability in roots and soils. Microbial diversity shifts across plant growth stages and microbial groups under stress. Enriched core taxa strengthen root microbial network stability under stress. These findings reveal how core microbes contribute to host drought resistance and health.

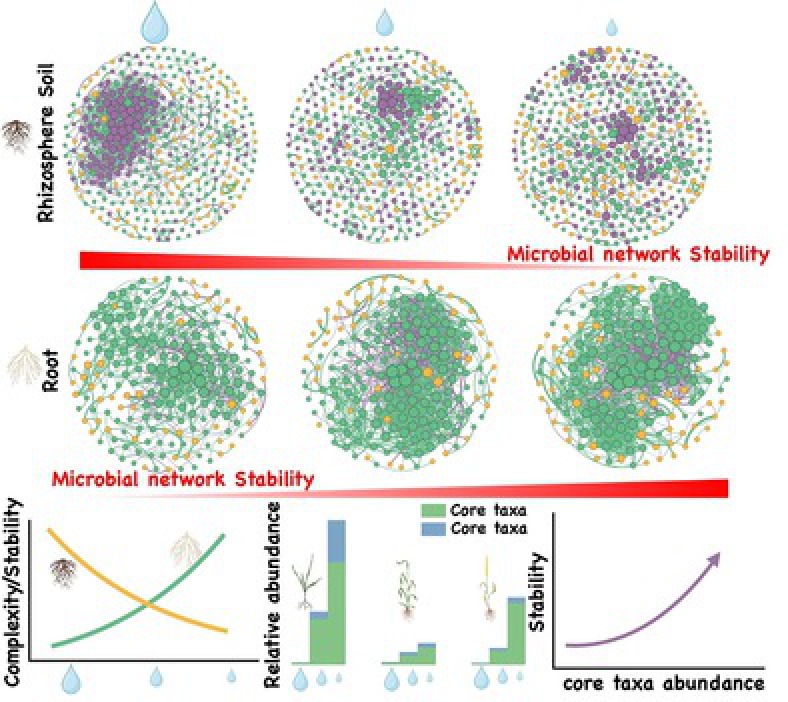

## 1 Introduction

Climate change has intensified the frequency and severity of drought stress, posing a major threat to global food security. The ability of crops to cope with environmental stresses, such as drought, can be partially supported by the microbiomes in their rhizosphere, roots and other compartments (Naylor and Coleman‐Derr 2018; de Vries et al. 2020; Trivedi et al. 2022). Drought stress promotes the enrichment of drought‐tolerant bacterial taxa, such as *Streptomyces*, which enhance plant resilience by producing osmolytes, polysaccharides, enzymes and plant hormones that support osmotic adjustment under drought stress (Yang et al. 2023; Liu et al. 2024; Li et al. 2025; Xiang et al. 2025). Fungal guilds, including endophytes, mycorrhizal fungi and decomposers contribute to plant drought responses through their diverse ecological functions. For example, pre‐flowering drought has been shown to increase the abundance of the fungal endophyte *Acremonium persicinum*, while reducing plant pathogenic fungi to improve crop drought resistance (Chen et al. 2025). Additionally, protists, as key microbial predators and drivers of nutrient cycling, can modulate microbial community composition and influence plant resource availability, potentially amplifying or buffering drought effects in plant‐associated environments (Geisen 2016a, 2016b; Gao et al. 2019).

Recent studies have underscored the significant impact of plant growth stages on the assembly of the plant microbiome (Zhang et al. 2018; Grady et al. 2019; Gao et al. 2020), due to the variation in plant physiological requirements and plant exudate composition over time (Chen et al. 2019; Zhao et al. 2021). The effects of drought stress also vary across plant growth stages, with different stages exhibiting different degrees of sensitivity. For example, the development of early‐stage sorghum root microbiome is delayed by drought, leading to higher levels of monoderm bacteria, which have thick cell walls and lack an outer cell membrane (Xu et al. 2018). These communities are shaped by a diverse set of microbial taxa with varying phylogenetic traits, which exhibit consistent longitudinal changes across growing seasons (Edwards et al. 2018). An age‐prediction model revealed that the bacteria of drought‐stressed plants exhibit developmental immaturity compared to unstressed plants, with microbial compositions reflecting juvenile and adult life stages (Edwards et al. 2018). Plant development stage drives microbiome succession, whereas drought can delay this process, potentially disrupting the plant‐microbiome synchrony required for plant health and stress adaptation.

Although previous studies have demonstrated how drought stress alters microbial diversity and community composition, the associations among plant‐associated microbiomes under drought stress remain underexplored despite their critical role in sustaining complex ecosystem functions (Deng et al. 2012). For instance, microbial associations have been closely linked to enhanced carbon degradation capacities and increased heterotrophic respiration (Yuan et al. 2021). Moreover, distinct plant symbionts can complement one another by supplying different limiting nutrients, thereby promoting greater plant productivity (Chen et al. 2025). Environment changes can reshape the co‐occurrence networks of soil microbial communities, significantly impacting the resilience and performance of hosts under stress (Zhou et al. 2011; Agler et al. 2016; Müller et al. 2016; Yuan et al. 2021). For example, warming significantly enhanced the network complexity, as indicated by increased size, connectivity, connectance, clustering coefficient, modularity and the number of keystone species compared to the ambient control. These networks also exhibited greater robustness, with stability positively associated with complexity (Yuan et al. 2021). Soil fungal networks are more resilient to drought stress than bacterial networks (de Vries et al. 2018). Previous studies on microbial co‐occurrence networks suggest that negative co‐occurrence patterns may enhance the robustness and stability of such networks under various disturbances (Coyte et al. 2015; Hernandez et al. 2021). Besides, most prior studies are focused on individual microbial species co‐occurrence networks (de Vries et al. 2018; Gao et al. 2020; Yuan et al. 2021), while the responses of inter‐kingdom microbial co‐occurrence networks to gradually intensified drought stress across rhizosphere soil and the root have received limited attention.

To clarify these patterns, we investigated how microbiome diversity, co‐occurrence network complexity and stability respond to increasing drought intensity. We assessed how varying drought intensities affect microbial diversity (prokaryotes, fungi and protists) in the rhizosphere and roots of wheat at tillering, jointing and ripening growth stages and examined the corresponding inter‐kingdom co‐occurrence networks. We selected the *Sceptre* cultivar, which is drought‐tolerant and a widely grown wheat variety in Southern and Western Australia. We hypothesised that (1) drought stress will differently affect the diversity of prokaryotes, fungi and protists in rhizosphere soil and root, with the most pronounced changes occurring at early growth stages; (2) drought stress will destabilise co‐occurrence networks in the rhizosphere soil due to the high sensitivity of protists and their weakened associations with other microbes and (3) drought stress will enhance the complexity and stability of the root microbial network through the enrichment of core taxa, which are more probably to benefit plants by increasing their drought tolerance.

## 2 Materials and Methods

### 2.1 Experimental Design

We collected soil (0–10 cm) for the glasshouse pot experiment in June 2022 from a broadacre crop paddock of the farm at the Dookie campus (36°33′S, 145°69′E) of The University of Melbourne, Victoria, Australia. The soil at this site is classified as Dermosol and the texture of the soil is silty loam with the following physicochemical properties: soil pH of 6.7 (1:5 water), soil organic matter 5.96% and total nitrogen of 0.21%. For the glasshouse pot experiment, the collected soil was air‐dried and sieved to a size of 4 mm mesh.

Wheat seeds (cultivar‐*sceptre*) were surface sterilised with 3% (v/v) sodium hypochlorite for 5 min and 70% (v/v) ethanol for 1 min. Five seeds were sown in each 5.4 L pot (23.0 cm height, 20.5 cm diameter) containing 5 kg soil. Three treatments with five replicates were established, including 75% water holding capacity (WHC) as the control (CK), 50% WHC representing moderate drought (MD) and 25% WHC representing severe drought (SD). In the first week, we maintained all pots at approximately 75% water holding capacity (WHC) to ensure germination. In the second week, we initiated drought stress treatments simultaneously for all plants. The soil dried to 50% WHC in about 3–4 days and to 25% WHC in about 7–8 days. Depending on the treatment, we maintained soil moisture at 75%, 50% or 25% WHC by adding water whenever it fell below the target level. We monitored pot weight regularly to guide watering (Figure S1A). In our study, drought stress treatment started on the same day. Soil and plant samples were then collected at three key growth stages (tillering, jointing and ripening stages), which reflect both the developmental stage of the plant and the duration of drought exposure.

We arranged all pots in a randomised block design in a controlled glasshouse, with a daily cycle of 16 h of light at 21°C and 8 h of darkness at 16°C. We collected soil and plant samples at the tillering, jointing and ripening stages to capture the combined effects of wheat development and prolonged drought exposure. The sampling methods of the bulk soil, rhizosphere soil and root followed the protocols with some modification (Edwards et al. 2015; Luo et al. 2017). Briefly, we carefully removed loosely adhering soil by hand and designated it as bulk soil, leaving approximately 1 mm of soil tightly attached to the root surface. We subsequently transferred roots into sterile 50‐mL Falcon tubes containing 30 mL of 1 × phosphate‐buffered saline (PBS) and shook them at 180 rpm for 15 min. The resultant suspension was filtered through a 100 μm sterile nylon mesh to eliminate root debris and large soil particles. The filtrate was then centrifuged at 5000 g for 10 min and the resultant pellet was collected as rhizosphere soil. The roots were washed sequentially with 80% ethanol and 1 × PBS, ground into a fine powder in liquid nitrogen and stored at −80°C until DNA extraction. The whole growth period of wheat spanned approximately 15 weeks, with the drought treatment applied from the second week onwards, lasting for 14 weeks (Figure S1A,S1B).

### 2.2 DNA Extraction and Amplicon Sequencing

We conducted DNA extraction following the protocols from previous studies (Edwards et al. 2015; Luo et al. 2017). We extracted DNA of bulk soil, rhizosphere soil and root samples by a DNeasy PowerSoil Kit (QIAGEN, VIC, Australia) following the instructions of the manufacturer. The concentration and purity of the extracted DNA were assessed using the NanoDrop ND2000c spectrophotometer (NanoDrop Technologies, Wilmington, DE, USA). The bacterial 16S rRNA gene, fungal internal transcribed spacer (ITS) region and eukaryotic 18S rRNA gene were amplified with the primer sets 341F/806R (Klindworth et al. 2013), ITS1F/ITS2R (Innis et al. 2012) and TAReuk454FWD1/TAReukREV3 (Stoeck et al. 2010), respectively.

We processed the amplicon sequencing data using USEARCH (v10) and QIIME2 (V.2023.2). We first merged the raw sequences and removed PCR primer sequences using Cutadapt (v4.2) (Nearing et al. 2018). We then filtered the merged sequences to exclude low‐quality, chimeric reads and those shorter than 200 bp. After chimera removal, we inferred exact sequence variants using the UNOISE algorithm implemented in USEARCH, which outputs zero‐radius OTUs (zOTUs) (Edgar 2010; Callahan et al. 2017). Taxonomic assignments for protistan sequences, derived from the eukaryotic 18S rRNA gene data, were performed using the Protist Ribosomal Reference (PR2) database v4.14.0 (Guillou et al. 2013). Taxonomic classification of prokaryotes and fungi was conducted against the SILVA database v138 (Pruesse et al. 2007) and the UNITE database v9.0 (Nilsson et al. 2019), respectively. We excluded prokaryotic sequences matching host mitochondria and chloroplasts from the analyses. Protists encompassed all eukaryotic taxa excluding fungi, invertebrates (Metazoa) and vascular plants (Streptophyta) (Delgado‐Baquerizo et al. 2020; Oliverio et al. 2020). We rarefied the zOTU tables using the vegan R package, following filtration, to 26,533 sequences for prokaryotes, 7992 sequences for fungi and 6869 sequences for protists, to standardise depth across all samples.

### 2.3 Microbial Co‐Occurrence Network Analysis

We constructed separate microbial co‐occurrence networks for each treatment using the rarefied microbial zOTU tables. To reduce the impact of rare zOTUs, only those zOTUs with a relative abundance greater than 0.01% were retained. Networks were constructed based on robust correlations, with Spearman's correlation coefficients (ρ) of > 0.8 or < −0.8 (*P*_FDR_ < 0.05) according to the recent studies (Weiss et al. 2016; Xiong et al. 2021; Qiao et al. 2024; Du et al. 2025; Liu et al. 2025). To assess the complexity of soil microbial networks, we computed various network characteristics, including the total number of nodes, number of links, relative modularity (RM) and average degree. Relative modularity indicates the degree to which a network is partitioned into distinct modules (Yuan et al. 2021). We generate network visualisations using the interactive Gephi software (Bastian et al. 2009).

We assess the stability of each network by the computation of natural connectivity and cohesion values. Robustness was employed to assess network stability by systematically removing nodes from the static network and observing the rate at which the inherent connectivity diminished (Peng and Wu 2016). Natural connectivity was introduced to characterize the redundancy of alternative pathways, a factor closely linked to the resilience of a network. We also tracked average degree under node removal to understand how basic topological properties change in parallel. This metric is defined as the ‘average eigenvalue’ of the graph's adjacency matrix:

(1) $$\bar{\lambda }=\frac{1}{n}\sum_{i=1}^{N}e^{\mathrm{\lambda i}}$$

where *N* is the number of nodes in a network and *λi* is the *i*th element of the set {*λ*1, *λ*2,…, *λN*}.

To assess the impact of drought stress on microbial network connectivity, we calculated microbial cohesion as previously described (Herren and McMahon 2017; Hernandez et al. 2021). For each sample, two cohesion values (positive and negative) were computed as the sum of significant positive or negative correlations between taxa weighted by taxon relative abundance:

(2) $$\text{Cohesion}=\sum_{i=1}^{n}\left({\text{abundance}}_{i}\times {\text{connectedness}}_{i}\right)$$

where *n* is the total number of taxa in a community. This cohesion index can be represented mathematically as the sum of the contribution of each of the taxa in the community.

Soil microbial community stability was assessed using the average variation degree (AVD) (Xun et al. 2021). A higher AVD value indicates lower microbiome stability, while a lower AVD value indicates higher microbiome stability. Network vulnerability is the maximum decrease in network efficiency when a single node is removed from the network. The vulnerability of each node quantifies its relative contribution to the overall efficiency of the network. The calculation method for vulnerability followed the approach as described previously (Yuan et al. 2021).

### 2.4 Identification of Core Taxa

To uncover potential core taxa within the network, we calculated the within‐module degree *z*‐score (*Zi*) for each node, which measures its connectivity to other nodes within the same module and the participation coefficient (*Pi*), which measures how its links are distributed among different modules. Module hubs (Zi>2.5, Pi≤0.62), connectors (Zi≤2.5, Pi>0.62) and network hubs (Zi≥2.5, Pi≥0.62) were referred to as keystone taxa (Deng et al. 2012). All other nodes were classified as peripherals. Additionally, we employed a SPEC‐OCCU plot (specificity‐occupy plot) to identify the potential specialist taxa within a community (Gweon et al. 2021). Different drought stress treatments were treated as distinct habitats, with a total of three habitats. The 500 most abundant zOTUs were selected for each habitat and their specificity and occupancy were calculated (Dufrêne and Legendre 1997). Specificity represents the ratio of average relative abundance of a zOTU in a habitat to the sum of the average relative abundances of the zOTU across all habitats. Higher specificity suggests that the taxa is more exclusive to a particular habitat compared with other habitats. Occupancy is characterized as the ratio of the number of samples in which the taxa is present to the total number of samples in a habitat. Higher occupancy suggests that the taxa is more prevalent in the habitat. These two metrics were employed as the axes in SPEC‐OCCU plot. In this study, we defined zOTUs with both specificity and occupancy higher than 0.7 as habitat specialists (Gweon et al. 2021; Qiao et al. 2024). The number of unique and shared zOTUs were calculated and visualised by the VennDiagram package (Chen and Boutros 2011).

### 2.5 Statistical Analyses

We analysed microbial alpha diversity using the function diversity from the vegan package (Oksanen et al. 2001). To assess beta diversity, we computed Bray‐Curtis distances between samples using the vegdist function with method = ‘bray’. We visualised changes in microbial community structures across different growth stages and drought stress treatments by performing non‐metric multidimensional scaling (NMDS) with the metaMDS function based on the Bray‐Curtis distance matrix (Oksanen et al. 2025). We used permutational multivariate analysis of variance (PERMANOVA) with the adonis2 function to evaluate differences in beta diversity across prokaryotes, fungi and protists in response to drought stress and wheat growth stages (Oksanen et al. 2025). We used variance partitioning analysis (VPA) to assess the relative importance of the explanatory variables: niches, growth stages and drought stress treatments using the vegan package (Oksanen et al. 2025). To evaluate the phylogenetic clustering of bacterial communities under different drought stress, we computed NTI (nearest‐taxon index) by the null model taxa. labels (999 randomisation) with the ses.mntd function of the picante package (Kembel et al. 2010). We identified the differentially abundant taxa or zOTUs in the roots under different drought stress treatments using the DESeq2 package (Love et al. 2014). A circular maximum likelihood phylogenetic tree was constructed by representative sequences of the abundant zOTUs with a relative abundance higher than 0.5%. We employed Itol.toolkit to create Interactive Tree Of Life format annotation files (Zhou et al. 2023) and used Interactive Tree Of Life to visualise the tree. Statistical analysis was performed using Wilcoxon or Kruskal–Wallis tests, followed by Dunn's post hoc pairwise comparisons with BH–FDR adjustment to determine whether the values were statistically significant among control (CK), moderate drought (MD) and severe drought (SD) treatments.

## 3 Results

### 3.1 Drought Stress Affected Microbial Diversity in Rhizosphere Soil and Root

**FIGURE 1:**
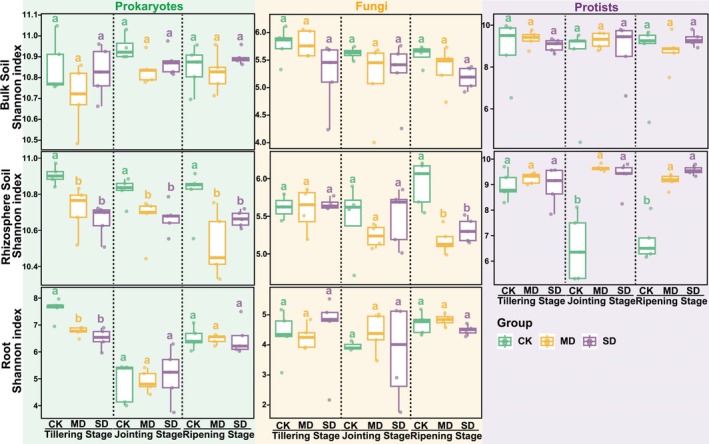
Drought stress impacts the alpha diversity of root‐associated microbiota at different growth stages. The boxes display the average Shannon diversity of microbiota (bulk soil, rhizosphere soil and root microbiota) under control (CK), moderate drought (MD) and severe drought (SD) treatments at different growth stages. Different letters indicate significant differences among treatments based on Dunn's post hoc test (BH–FDR adjusted) following the Kruskal–Wallis test.

The diversity of microbiota was significantly influenced by drought stress, but these effects were dependent on microbial kingdoms, plant niches and growth stages (Figure 1, Figure S1C). In bulk soil, drought stress did not significantly affect microbial alpha diversity in any microbial kingdoms or growth stages. In rhizosphere soil, responses to drought stress varied across microbial kingdoms. Specifically, prokaryote alpha diversity (Shannon index) significantly decreased under drought stress at the tillering stage, but fungal and protist diversity remained unchanged. Drought stress significantly reduced fungal diversity only at the ripening stage, while an opposite trend was found for protists. In roots, prokaryotic diversity was significantly reduced at the tillering stage under drought stress, but no significant changes were observed at the jointing and ripening stages. Fungal diversity showed no significant changes under drought stress. In addition, regardless of drought stress, the jointing stage has the lowest prokaryotic and fungal alpha diversity compared with the tillering and ripening stages in the root, but this pattern was not observed in either rhizosphere or bulk soil (Figure S1C).

### 3.2 Drought Stress Affected Microbial Community Compositions in Rhizosphere Soil and Root

**FIGURE 2:**
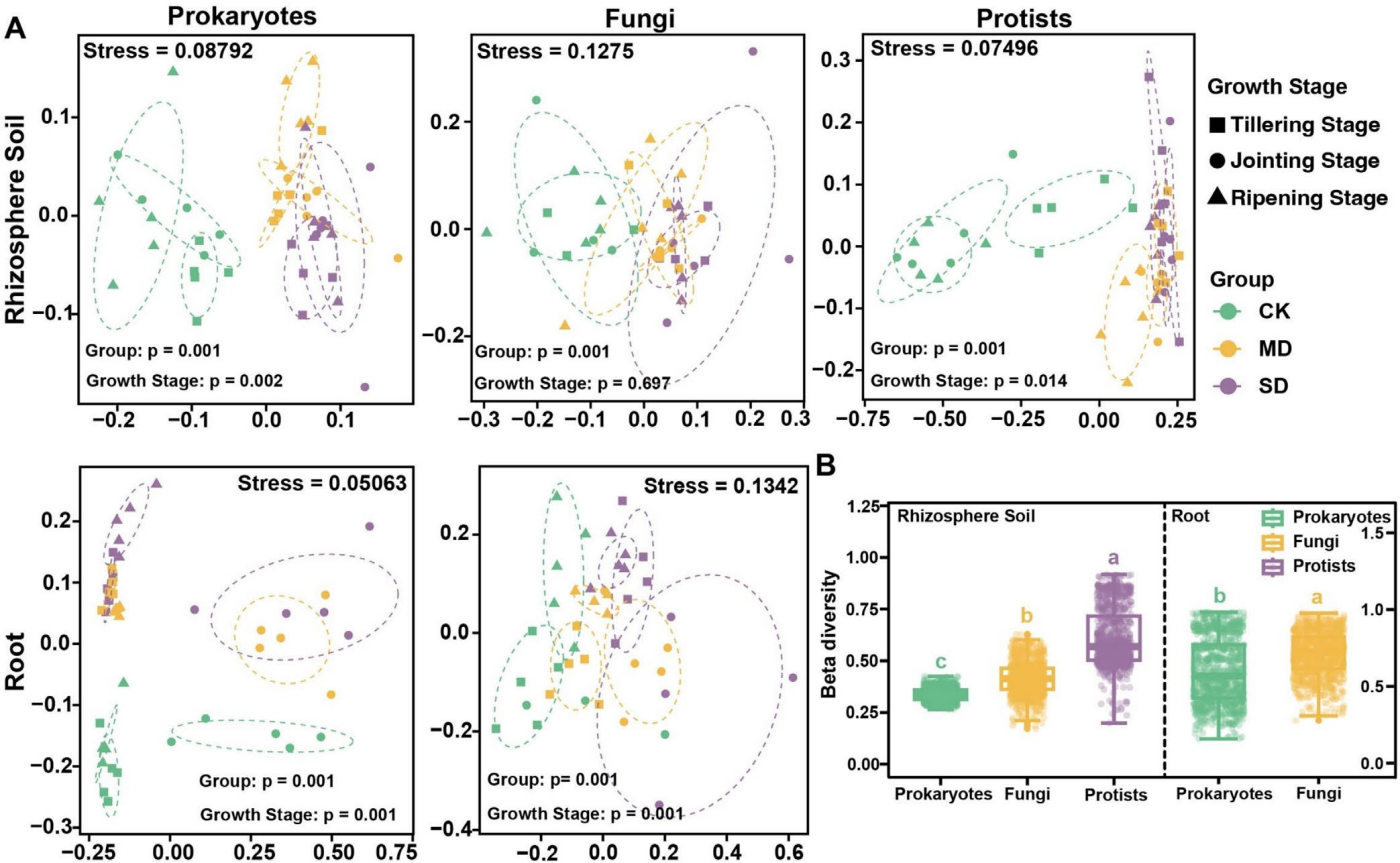
(A) Drought stress impacts the beta diversity of root‐associated microbiota in different growth stages. Non‐metric multidimensional scaling (NMDS) based on Bray–Curtis dissimilarity shows the dissimilarity of root‐associated microbiota (rhizosphere soil and root microbiota) under control (CK), moderate drought (MD) and severe drought (SD) treatments in different growth stages. *p* values for PERMANOVA were obtained by permutation tests (adonis2; 999 permutations). *p < 0.05; **p < 0.01; NS: not significant. (B) Differences in beta‐diversity (Bray–Curtis) among microbial groups were tested using the Kruskal–Wallis test in the rhizosphere (prokaryotes, fungi, protists) and Wilcoxon test in the root (prokaryotes, fungi); post hoc pairwise comparisons were performed using Dunn's test with the BH–FDR adjustment (rhizosphere).

The NMDS analysis revealed that samples from different drought stress treatments formed distinct clusters, with significant differences confirmed by the PERMANOVA test (Figure 2A, Figure S2B). Among the microbial groups in the rhizosphere, protists exhibited the greatest separation under drought stress (*R* ^2^ = 0.330, *F*‐value = 11.007), followed by fungi (*R* ^2^ = 0.202, *F*‐value = 4.528) and prokaryotes (*R* ^2^ = 0.167, *F*‐value = 4.358) (Figure 2B, Figure S2B), indicating that rhizosphere protist community composition was more sensitive to drought. In roots, fungal communities showed the largest separation, followed by prokaryotes (Figure 2B, Figure S2B). Significant differences were observed across prokaryotic, fungal and protist communities in both rhizosphere soil and roots under different drought stress treatments.

Variance partitioning analysis (VPA) was employed to quantify the contributions of soil/root compartments (niches), growth stages (stage) and drought stress (stress) to variations in the three microbial groups. The Venn diagram revealed that niche explained 46%, 28% and 19% of the variation in prokaryotic, fungal and protistan communities, respectively and drought stress had greater explanation than stages (Figure S2A).

### 3.3 Drought Stress Affected Microbial Co‐Occurrence Network Complexity and Stability

**FIGURE 3:**
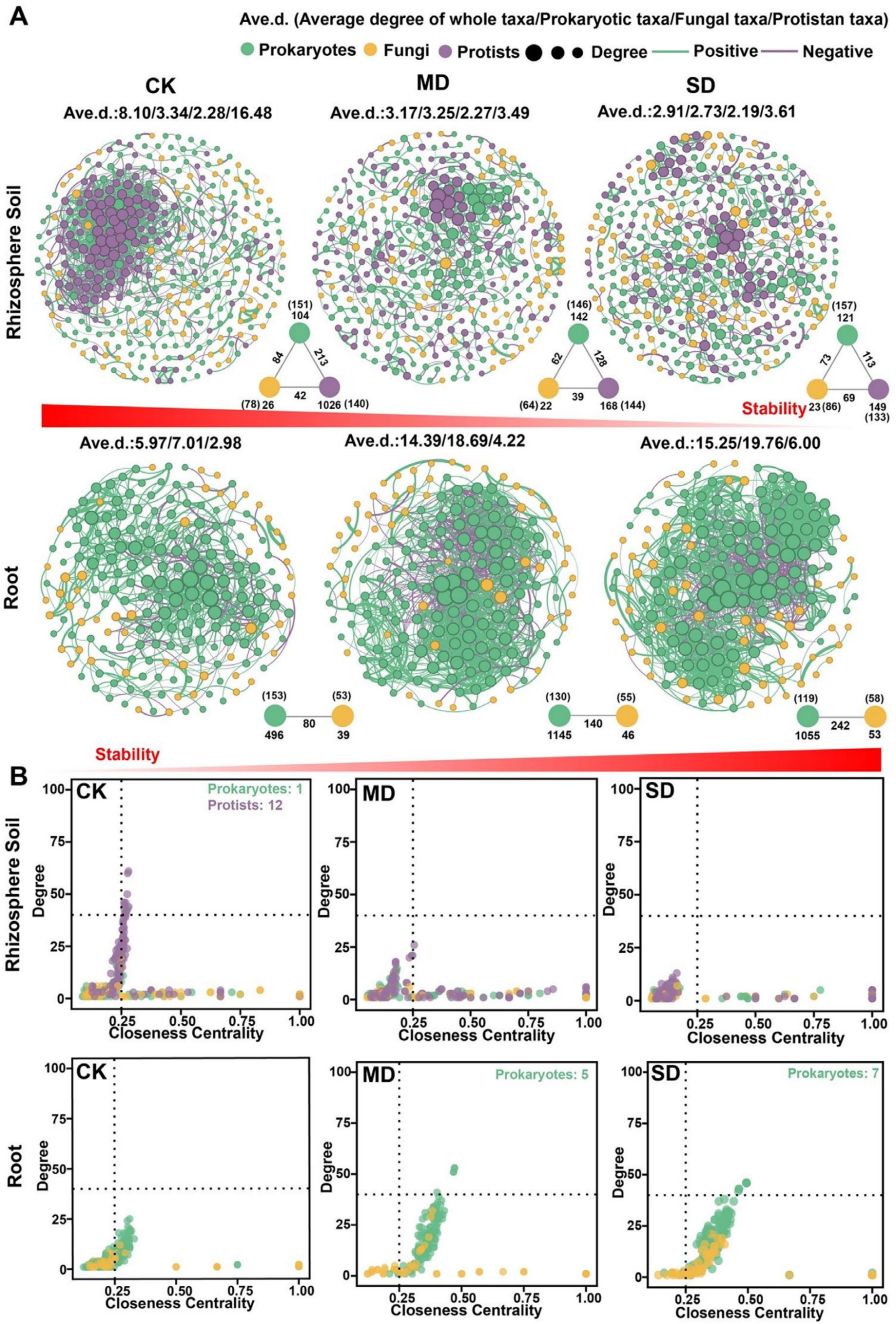
(A) Inter‐kingdom co‐occurrence networks in the rhizosphere soil and root under control (CK), moderate drought (MD) and severe drought (SD) treatments. The numbers in the triangle or line segment without parentheses indicate the number of intra‐kingdom/inter‐kingdom edges, while the numbers in parentheses represent the number of nodes within each microbial group (prokaryotes, fungi and protists). (B) Comparison of node‐level topological features (degree and closeness centrality) among microbiomes under CK, MD and SD treatments. Each point in the scatterplot represents a node (zOTU) and is plotted based on its calculated degree (*y*‐axis) and closeness centrality (*x*‐axis).

**FIGURE 4:**
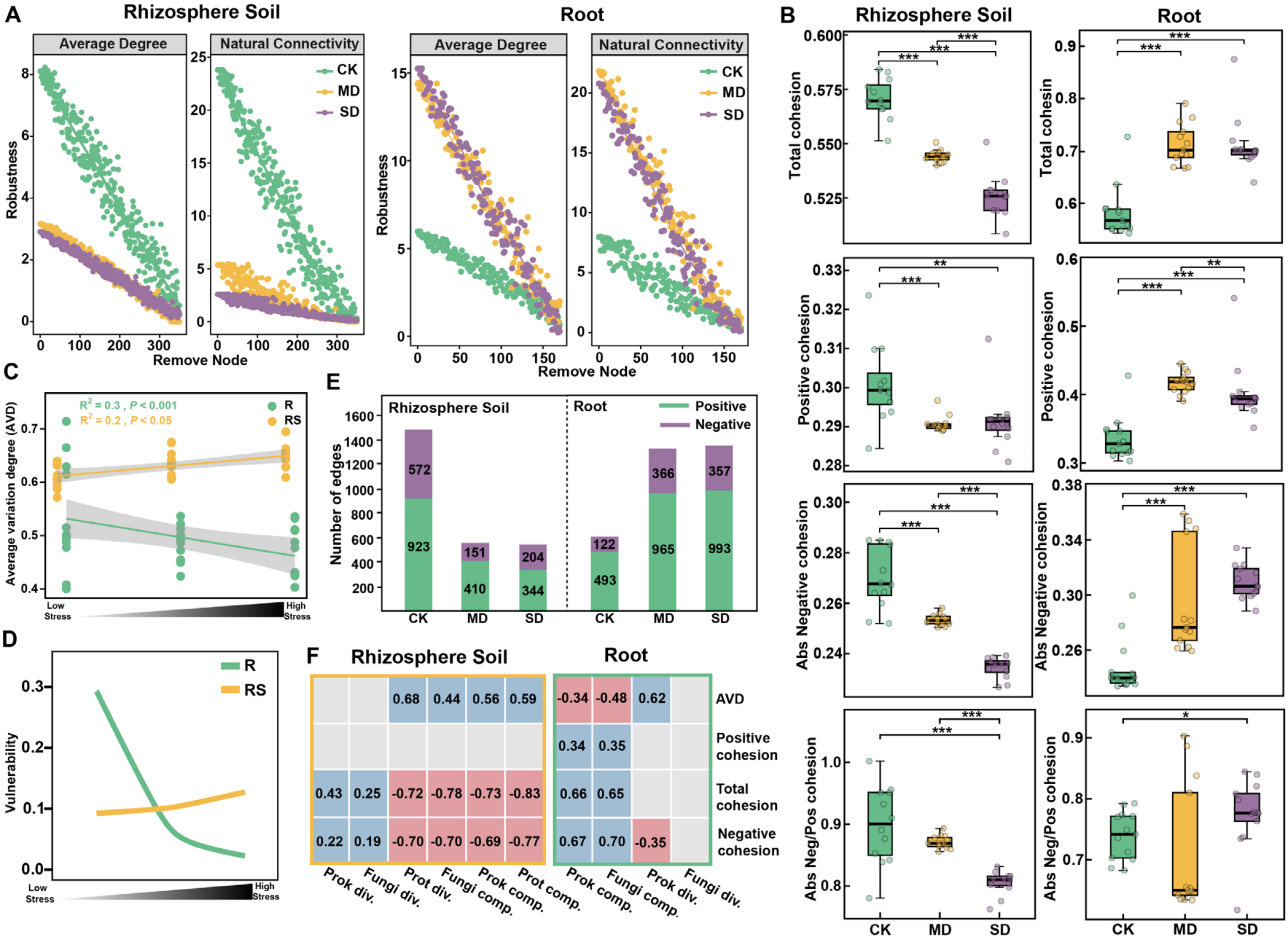
(A) Changes in robustness (average degree and natural connectivity) under control (CK), moderate drought (MD) and severe drought (SD) treatments in rhizosphere soil and root. A given number of nodes were randomly removed from the network and the natural connectivity was calculated. This process was repeated across a range of node removal levels (from 0 to X node) and the resulting decline in natural connectivity was used to indicate network robustness. Changes in average degree were tracked under node removal to evaluate shifts in basic topological properties. (B) Changes in total cohesion, positive cohesion, the absolute value of negative cohesion and the ratio of absolute negative cohesion to positive cohesion of rhizosphere soil and root under CK, MD and SD treatments. The cohesion values for each drought stress include samples from three different growth stages. Different asterisks indicate a significant difference at *p < 0.05, **p < 0.01, ***p < 0.001 based on Wilcoxon test. (C) The relationship between drought stress and average variation degree (AVD) of the microbial communities in the rhizosphere soil and root. The replicates shown under each drought stress include samples from three different plant growth stages. (D) Network vulnerability measured by maximum node vulnerability for different treatments (CK, MD and SD) in the rhizosphere soil and root. (E) The number of positive edges and negative edges in rhizosphere soil and root under CK, MD and SD treatments. (F) Pearson correlations between microbial diversity and network stability indices in rhizosphere soil (boxed in yellow) or root (boxed in green). Significant correlations (*p* < 0.05) are shown, with pink for negative correlations and blue for positive correlations. Not significant correlations are marked in grey. Numbers inside cells are correlation coefficients.

To compare how drought stress affected the complexity and stability of rhizobacterial and root co‐occurrence networks, six networks were constructed. Our results revealed shifts in microbial interkingdom network patterns for rhizosphere soil and roots across drought stress treatments (Figure 3A). Specifically, network connectivity (i.e., network degree) decreased in the rhizosphere soil with increasing drought stress. Connectivity declines were observed for prokaryotes, fungi and protists and the most pronounced decline, 78.09%, was observed in protists. In contrast, network connectivity in roots increased with increasing drought stress, particularly for prokaryotes, which showed the most significant increase of 181.88% (Figure 3A). Consistent with these connectivity changes, edge counts further captured the drought‐driven shifts in network complexity. Specifically, the number of edges decreased with increasing drought stress in the rhizosphere soil, whereas the opposite trend was observed in roots (Figure 3A). Furthermore, we also examined positive and negative edges and found that their responses to increasing drought differed between the rhizosphere soil and root (Figure 4E).

We defined ‘network hubs’ as nodes with high closeness centrality (> 0.25) and degree (> 40). In the rhizosphere soil, 13 network hubs (12 protists, 1 prokaryote) were identified in the control treatment, but no network hubs were found under moderate and severe drought conditions (Figure 3B). The prokaryotic hub taxa belonged to *Cyanobacteria*, while protist hubs mainly consisted of *Conosa* and *Chlorophyta*. In root networks, 5 prokaryotic hubs were identified in the moderate drought stress and 7 prokaryotic hubs in the severe drought stress (Figure 3B). The prokaryotic hubs were mainly from *Actinobacteria* and *Proteobacteria*.

Analysis of robustness and cohesion indicated that the stability of microbial networks decreased under drought stress in the rhizosphere soil but increased in the root (Figure 4). In the rhizosphere soil, robustness was consistently higher in the control treatment compared with moderate and severe drought stress treatments when nodes were randomly removed. Conversely, in the root, robustness declined more rapidly when control nodes were randomly removed compared with moderate and severe drought stress nodes (Figure 4A). Additionally, the ratio of absolute negative cohesion to positive cohesion decreased under drought stress in the rhizosphere soil, suggesting that positive associations between taxa became more dominant. In contrast, this ratio increased under drought stress in the root, suggesting that negative associations between taxa were more prevalent. Total cohesion (sum of absolute negative and positive cohesion) also decreased in the rhizosphere soil but increased in roots with increasing drought stress. This indicates a reduction in microbial community stability in the rhizosphere soil, but an increase in stability in the root (Figure 4B). The decline in network stability in the rhizosphere soil, represented by reduced total cohesion, resulted from a decrease in both positive and absolute negative cohesion magnitudes with increasing drought stress. Specifically, absolute negative cohesion in the rhizosphere soil declined by 13.12% and positive cohesion by 3.16% between the control and severe drought stress treatments. Conversely, in the root, absolute negative cohesion increased by 25.13% and positive cohesion by 19.96% between the control and severe drought stress treatments. Changes in absolute negative cohesion were greater than changes in positive cohesion in both rhizosphere soil and root along the gradient, indicating that negative co‐occurrence was a stronger driver of total cohesion results.

The AVD values significantly increased with increasing drought stress in the rhizosphere soil, but decreased in the root, indicating that microbial community stability decreased in the rhizosphere soil but increased in the root under drought stress (Figure 4C). Similarly, network vulnerability increased in the rhizosphere soil as drought severity intensified, while the opposite trend was observed in the root (Figure 4D). Significant correlations were observed between alpha diversity and stability metrics. In the rhizosphere soil, network stability was positively correlated with prokaryotic diversity and negatively correlated with protistan diversity, but a negative correlation with prokaryotic diversity was observed in the root (Figure 4F).

We found that prokaryotes dominated in maintaining root microbial network stability under drought stress (Figure 3) and therefore, we constructed a microbial network focusing solely on prokaryotes (Figure S3). Specifically, prokaryote network properties, including the number of nodes, the number of edges, average degree, modularity and node degree were enhanced as drought stress increased (Figure S3A,B,C). Moreover, to evaluate whether drought stress altered the phylogenetic structure of prokaryotic communities in the root, we quantified the Nearest Taxon Index (NTI), which indicates the degree of phylogenetic clustering (higher NTI) or dispersion relative to a null expectation. Although mean NTI did not differ significantly among CK, MD and SD treatments, NTI tended to be higher under SD (mean NTI > 2), suggesting a stronger tendency toward phylogenetic clustering under SD stress (Figure S3D). Collectively, drought stress enhanced the stability of the root microbial network but reduced the stability of the rhizosphere soil microbial network.

### 3.4 Core Microbial Taxa Under Drought Stress

**FIGURE 5:**
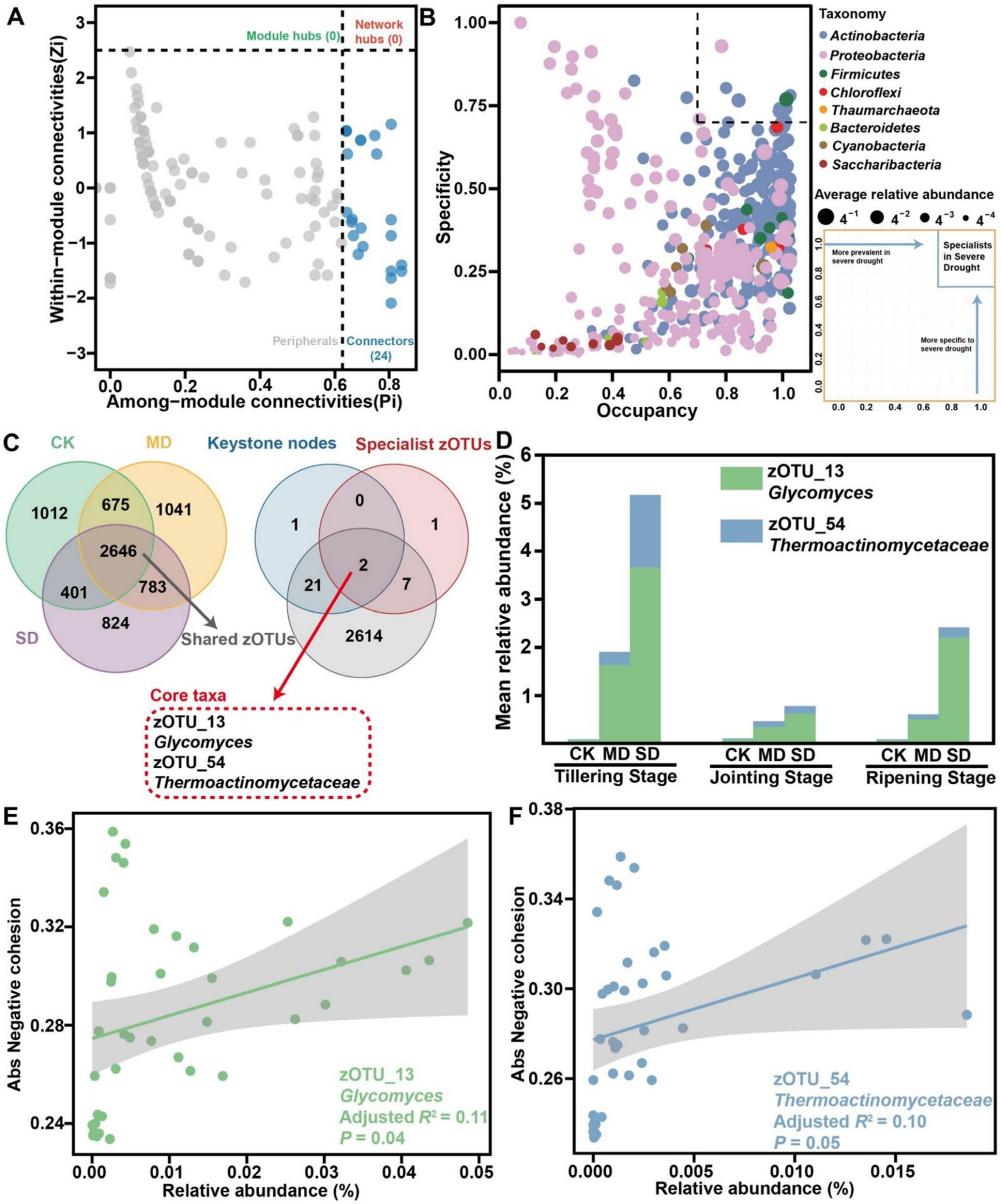
Identification of core taxa in the root. (A) The Zi‐Pi method to reveal keystone nodes of root microbial network under severe drought stress. (B) The SPEC‐OCCU plots of severe drought stress treatment show 500 most abundant zOTUs in each habitat type. (C) Screening for core taxa based on ‘Shared zOTUs’, ‘Specialist zOTUs’ and ‘Keystone nodes’. (D) The relative abundance of core taxa under control (CK), moderate drought (MD) and severe drought (SD) treatments in different growth stages. (E) Pearson correlations between the absolute negative cohesion and the relative abundance of zOTU_13 *Glycomyces*. (F) Pearson correlations between the absolute negative cohesion and the relative abundance of zOTU_54 *Thermoactinomycetaceae*.

We further investigated the root prokaryotic communities to identify the potential core taxa and their contributions to network stability. Several analyses were employed to determine core taxa: (1) network keystone nodes defined by calculating the within‐module connectivity (*Zi*) and among‐module connectivity (*Pi*); (2) taxa with high specificity and occupancy values (> 0.7); and (3) zOTUs that were simultaneously identified as keystone taxa, specialist zOTUs and zOTUs shared by root prokaryotes across all three treatments, as illustrated by their overlap in the Venn diagram. Our results revealed that a majority of nodes under severe drought stress network were classified as peripherals, suggesting that their connections were largely within their respective modules. A total of 24 connectors were identified as keystone nodes in the SD stress network that originated from three phyla: 12 from *Actinobacteria*, 1 from *Firmicutes* and 11 from *Proteobacteria* (Figure 5A, Table S1).

Additionally, zOTUs with specificity and occupancy above 0.7 were identified as specialists. Ten specialist zOTUs were identified, including 7 from *Actinobacteria*, 1 from *Firmicutes* and 2 from *Proteobacteria* (Figure 5B, Figure S4). Notably, zOTU_13 (*Glycomyces*) belongs to *Actinobacteria* and zOTU_54 (*Thermoactinomycetaceae*) belonged to *Firmicutes* and were identified as both keystone nodes and specialists (Figure 5C). zOTU_13 and zOTU_54 were shared across all three drought stress treatments. The relative abundances of these core taxa (especially zOTU_13 and zOTU_54) were higher in moderate and severe drought stress treatments than in the control treatment across three growth stages, especially at the tillering stage (Figure 5D). To further validate the importance of these core taxa in maintaining the stability of root microbial networks, we conducted a correlation analysis. The results showed that the relative abundances of these two core taxa were positively correlated with negative cohesion (Figure 5E,F).

**FIGURE 6:**
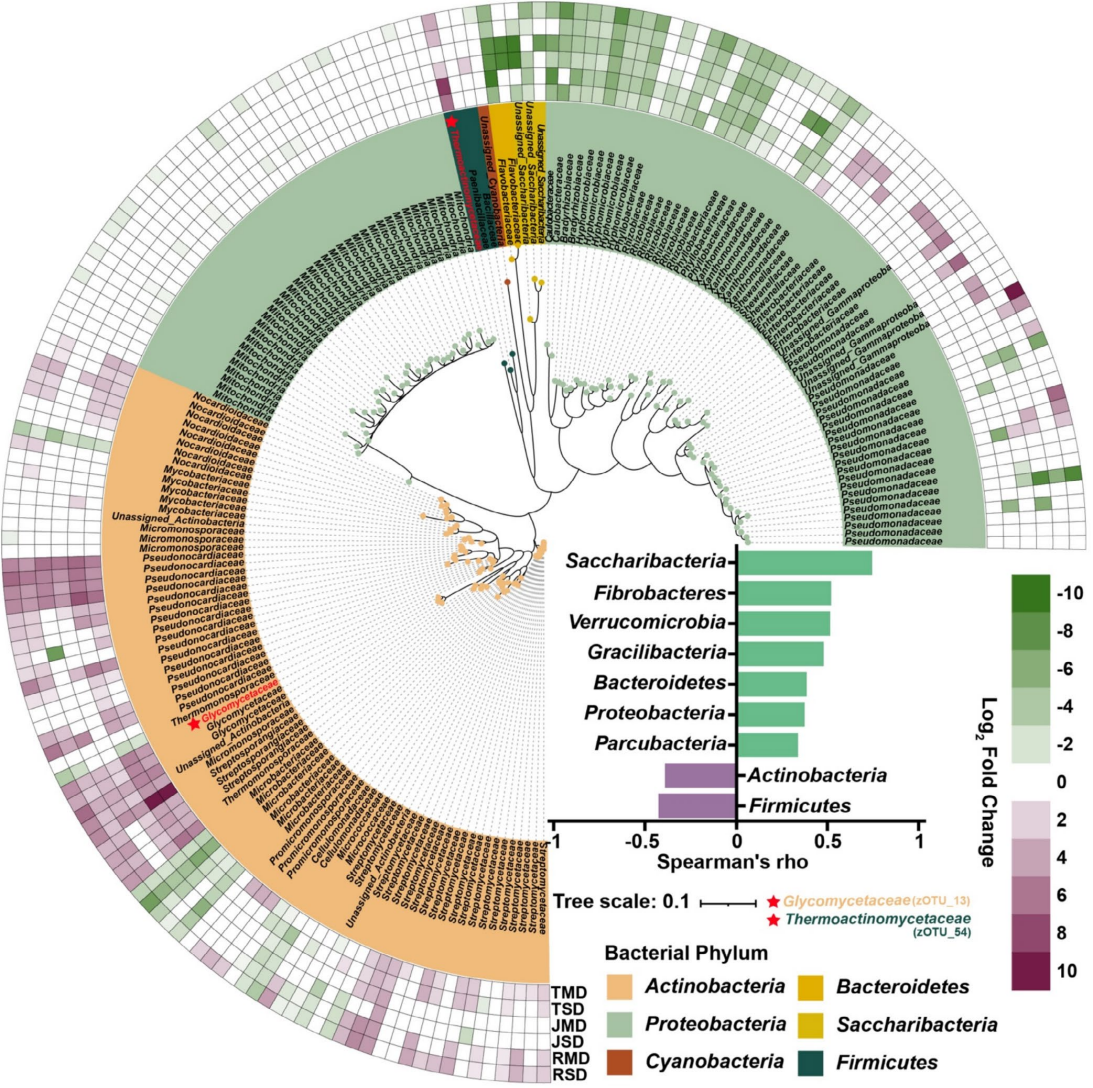
Phylogenetic relationships and distribution of 183 dominant prokaryotic zOTUs under drought stress treatments across different growth stages. TMD moderate drought at tillering stage, TSD severe drought at tillering stage, JMD moderate drought at jointing stage, JSD severe drought at jointing stage, RMD moderate drought at ripening stage, RSD severe drought at ripening stage. Log2‐fold change, as calculated by DESeq2 analysis, of significantly decreasing and increasing bacterial zOTUs in severe drought and moderate drought stress treatments compared with the control treatment is represented by the green‐to‐purple colour gradient. The affiliation of zOTUs at the phylum is indicated by different colours on the tips of phylogenic tree and the internal ring. The prokaryotic phylum whose abundance are the most positively or negatively correlated with the drought stress gradient (all correlations with *p* < 0.05 after BH‐FDR correction).

We quantified the changes in root prokaryotes in response to two levels of drought and growth stages to identify which groups were most influenced by the drought stress treatments. In general, with increasing drought stress, certain microbial groups become enriched, including core taxa (Figure 6). *Actinobacteria* (Spearman's *ρ* = −0.390, *P*_FDR_ < 0.01) and *Firmicutes* (Spearman's *ρ* = −0.425, *P*_FDR_ < 0.01) which were positively associated with increasing drought stress (Figure 6, Table S2). The abundance of *Glycomyces* (*Actinobacteria*, zOTU_13) increased from 0.0716% in the control treatment to 3.638% in the severe drought stress at the tillering stage. Similarly, the abundance of *Thermoactinomycetaceae* (*Firmicutes*, zOTU_54) increased from 0.003% in the control treatment to 1.489% in the severe drought stress at the tillering stage (Figure 6, Table S3).

## 4 Discussion

### 4.1 Microbial Diversity Responses to Drought Differ Across Plant Growth Stages and Microbial Groups

Our study investigated changes in alpha diversity across growth stages under different drought stress levels. We observed a consistent decline in root microbiome diversity from the tillering stage to the jointing stage under CK, MD and SD (Figure S1C). This stage‐dependent decline likely reflects stronger host filtering as roots develop. Developmental transitions can alter root physiology and immune gating and often reshape both the amount and composition of root exudates. Together, these changes modify resource availability and selectively favour well‐adapted taxa, ultimately reducing alpha diversity (Sasse et al. 2018; Zhalnina et al. 2018). For example, *Nitrospira* has been reported to accumulate in rice roots at early stages and remain abundant at the late stages, indicating that the host may actively recruit these microbes to regulate nitrogen availability for plant growth (Zhang et al. 2018; Liu et al. 2019).

Interestingly, both prokaryotic and fungal diversity in roots increased at the ripening stage under CK, MD and SD (Figure S1C). A plausible explanation is that during reproductive development, enhanced rhizodeposition (including root senescence, sloughed cells and exudation) broadens and diversifies the substrate supply, thereby creating additional niches that support a more diverse microbial assemblage. This pattern is consistent with developmentally programmed shifts in root exudate chemistry that shape microbiome assembly and promote microbial recruitment into the root (Zhalnina et al. 2018). This recruitment of microbes may support plant growth, especially under stress conditions (Li et al. 2016; Liu et al. 2019). The drought‐enrichment of *Glycomyces* and *Thermoactinomycetaceae* (Figure 5D), particularly at the tillering stage, may further contribute to reduced root alpha diversity by increasing dominance of drought‐adapted ‘monoderm’ lineages (thicker cell walls, spore formation and stress tolerance), which are repeatedly observed to rise under drought and can competitively exclude drought‐sensitive taxa (Figure 1) (Xu et al. 2018). Moreover, drought effects are often stronger during early microbiome assembly; drought has been shown to delay microbiome development and generate ‘developmentally immature’ root communities, whereas later‐stage communities tend to be more stabilised, potentially buffering compositional shifts under stress (Xu et al. 2018). In contrast, rhizosphere protists exhibited higher alpha diversity (Shannon index) under drought (Figure 1), particularly at the jointing and ripening stages and showed the greatest drought–control separation in beta diversity (Figure 2, Figure S2B). Because the Shannon index is sensitive to both richness and evenness, this pattern likely reflects increased community evenness rather than a simple increase in richness. Drought reduced phototrophic protists (e.g., *Chlorophyta*) while increasing multiple non‐phototrophic lineages, thereby weakening dominance by a few taxa and redistributing relative abundances more evenly (Figure S5). In addition, many free‐living protists act as key microbial predators and can track changes in bacterial prey availability. Drought stress and ripening stages may increase the spatial patchiness of bacterial ‘hotspots’ driven by rhizodeposition, potentially promoting the coexistence of multiple predatory protist taxa and further elevating diversity (Gao et al. 2019). As many soil protists depend on thin water films for activity and dispersal, drought‐driven reductions in soil water availability can strongly restructure protistan communities by altering water‐film connectivity and dispersal, thereby amplifying compositional turnover and enhancing drought–control separation in beta diversity (Geisen et al. 2014). Collectively, these results suggest that the observed diversity trajectories across microbial groups and plant development are shaped by stage‐dependent host filtering and rhizodeposition, together with drought‐driven selection for stress‐tolerant taxa and moisture‐sensitive protist turnover in the rhizosphere.

### 4.2 Drought Stress Destabilises Microbial Stability in the Rhizosphere Soil but Strengthen the Stability in the Root

The ecological stability of the plant microbiome is crucial for maintaining plant health (Gao et al. 2021; Qiao et al. 2024). Our results revealed that microbial inter‐kingdom network patterns shifted significantly in response to drought stress in both rhizosphere soil and roots (Figure 3). In the rhizosphere soil, increased drought stress led to networks with fewer nodes, edges and network hubs, indicating a decrease in network complexity. In contrast, the root microbial network displayed an opposite trend, with increased complexity under drought stress (Figures 3, 4, S3). We suggest that these opposing patterns are largely driven by compartment‐specific shifts in the dominant network hubs. In the rhizosphere, protists often act as cross‐trophic ‘connectors’ linking multiple microbial kingdoms through predation and other interactions, yet they are among the most moisture‐sensitive components of the rhizosphere microbiome. Drought‐driven reductions in soil water availability can disrupt water‐film connectivity and intensify environmental filtering and dispersal limitation, which may sharply reduce protist hubs and weaken cross‐kingdom associations, thereby simplifying the network. By contrast, the root endosphere provides a more buffered microenvironment and is strongly shaped by host filtering via physical barriers, immune gating and nutrient inputs. Under drought, roots may enrich stress‐tolerant prokaryotic lineages and the increase in prokaryotic hubs may strengthen inter‐kingdom co‐occurrence and network organisation, resulting in higher connectivity and complexity. High microbial network complexity has been suggested to contribute to ecosystem resilience, as intricate microbial co‐occurrences can stabilise communities against environmental disturbances (Yuan et al. 2021). Complex networks facilitate redundancy in functional roles, meaning multiple microbes can perform similar ecological functions, which enhances the ecosystem's ability to maintain functionality under stress (Coyte et al. 2015; Hernandez et al. 2021; Qiao et al. 2024). Understating how the complexity of microbial inter‐kingdom network is impacted by the drought stress has important implications for maintaining ecosystem services and agriculture productivity.

Although direct measurement of microbial interactions in a community remains currently unfeasible, cohesion analysis provides insights into associations among taxa driven by positive and negative correlations, which could simulate microbial co‐occurrences in a community by calculating positive and negative cohesion (Herren and McMahon 2017). This method has been used in many studies, for example, community coalescence by mixing more healthy soils resulted in higher cohesion and network stability as well as complexity (Qiao et al. 2024). Positive relationships represent niche overlap or positive correlations between taxa, while negative relationships suggest divergent niches or competition. The ratio of absolute values of negative and positive cohesion can serve as an indicator of network stability (Herren and McMahon 2017; Hernandez et al. 2021). In a previous study, plant‐microbial mutualists are some of the taxa most positively associated with the stress gradient across both prokaryotes and fungi, for example, the abundance of ectomycorrhizal fungi increased in a stress environment, suggesting that positive associations are relatively destabilising (Hernandez et al. 2021). We found that in the rhizosphere soil, the ratio of absolute value of negative cohesion decreased under drought stress, indicating a destabilisation of the microbial network in the soil environment. By contrast, the increasing ratio in the root samples represents improving the stability of the network under drought stress (Figure 4B). In addition, robustness (the resistance to node loss), AVD (average variation degree) and vulnerability have been proposed for the assessment of network stability (Xun et al. 2021; Yuan et al. 2021). In our results, the robustness tended to improve with increasing drought stress in the root, however, AVD and vulnerability showed the opposite trend (Figure 4A,C,D). While these metrics do not directly capture biotic interactions, they provide valuable perspectives on the organisational features and potential resilience of microbial communities. A stable microbial network is generally associated with sustained ecosystem functions such as nutrient cycling, which are essential for plant productivity and soil health in both agricultural and natural systems (Yuan et al. 2021; Qiao et al. 2024). Additionally, stable microbial networks facilitate beneficial correlations among microbes, enhancing the community's capacity to resist pathogen invasions and environmental stress (Hernandez et al. 2021).

### 4.3 Core Taxa Strengthen the Root Microbial Network Stability Under Drought Stress

Changes in network complexity and stability can often be driven by shifts in the abundance of key community members (Montesinos‐Navarro et al. 2017; Yuan et al. 2021). Microbial core taxa represent critical components of microbial communities, as their presence, absence or changes in abundance can substantially reshape microbiome structure and function (Banerjee et al. 2018; Toju et al. 2018). By integrating the Venn, Zi‐Pi and SPEC‐OCCU plots, we identified two core taxa (zOTU_13 and zOTU_54) that increased in relative abundance under drought stress (Figure 5). These core taxa were assigned to *Glycomyces* (*Actinobacteria*) and *Thermoactinomycetaceae* (*Firmicutes*), two Gram‐positive groups that are frequently reported to be drought‐favoured and to support plant performance, potentially via osmotic/oxidative stress mitigation and competitive exclusion of opportunistic microbes (Xu et al. 2018; Xu and Coleman‐Derr 2019). Mechanistically, drought can reprogram plant physiology and root exudation, generating a metabolite‐driven ‘cry‐for‐help’ signal that may selectively recruit beneficial microbes to the rhizosphere soils and roots (Li et al. 2025; Xiang et al. 2025). For example, a recent multi‐omics study showed that drought‐stressed wheat enriches osmoprotective metabolites (notably 4‐oxoproline) in the rhizosphere. Notably, 4‐oxoproline was positively associated with a drought‐enriched *Streptomyces* taxon and could directly stimulate its growth, supporting metabolite‐mediated recruitment of beneficial *Actinobacteria* under drought stress (Li et al. 2025). In addition, drought‐responsive phytochemicals (e.g., jasmonic acid and pipecolic acid) have been correlated with drought‐enriched microbial assemblages across plant compartments, consistent with a metabolite‐filtering mechanism that shapes drought‐adapted microbiomes (Xiang et al. 2025). Similar host‐driven selection has been reported in other contexts, for instance, maize may select *Glycomycetaceae* (*Actinobacteria*) linked to enhanced phosphorus acquisition during early growth in compacted soils (Zhang et al. 2023). Moreover, targeted disruption of protective *Firmicutes* and *Actinobacteria* in the tomato rhizosphere increased the incidence of bacterial wilt disease (Lee et al. 2021). Notably, in our study, negative cohesion increased with the relative abundance of core taxa, suggesting that these drought‐enriched core members may contribute to reinforcing community stability under stress, potentially by strengthening biotic constraints on opportunistic taxa. All these findings support a framework in which drought‐induced plant metabolic adjustments may help recruit and maintain beneficial core taxa, thereby reinforcing microbial network stability and ultimately promoting crop resilience under drought stress.

## 5 Conclusions

This study systematically evaluated the effects of different drought stress on the microbial diversity and network stability in the rhizosphere soil and plant root of a drought tolerant wheat cultivar. The effects of drought stress on microbial diversity depended on microbial groups, plant niches and growth stages. Additionally, drought stress decreased microbial network stability in the rhizosphere soil but increased microbial network stability in the root. Furthermore, core taxa (*Glycomyces* and *Thermoactinomycetaceae*) were enriched and strengthened the root microbial network stability under drought stress. This paves the way for understanding the mechanisms that core taxa contribute to host resistance and health in the stressed environment. Overall, these findings greatly enhance our fundamental understanding of crop‐microbiome correlations under drought stress and offer essential insights for future research on synthetic communities and for developing microbiome‐based tools to improve sustainable crop protection and agricultural productivity under stress.

## Author Contributions

**Keren Wu:** conceptualization, methodology, software, data curation, investigation, validation, formal analysis, visualization, writing – original draft, writing – review and editing. **Hang‐Wei Hu:** conceptualization, writing – review and editing. **Dorin Gupta:** conceptualization, writing – review and editing. **Yuan Li:** writing – review and editing. **Zi‐Yang He:** writing – review and editing. **Feng Wang:** writing – review and editing. **Ji‐Zheng He:** conceptualization, funding acquisition, writing – review and editing, project administration, supervision, resources.

## Funding

This work was supported by the Australian Research Council (IH200100023) and Department of Agriculture, Fisheries and Forestry, Australian Government (4‐H808GTH).

## Conflicts of Interest

The authors declare no conflicts of interest.

## Supporting information


**Figure S1:** (A, B) A schematic diagram of experiment process. The percentage of soil water holding capacity (WHC) under control (CK), moderate drought (MD) and severe drought (SD) treatments. The drought stress treatments began from the second week. The soils and plants were harvested in the tillering stage, jointing stage and ripening stage (the black arrows). (C) The changes in the alpha diversity of prokaryotes, fungi and protists across the bulk soil, rhizosphere soil and root at three different growth stages under control (CK), moderate drought (MD) and severe drought (SD) treatments. Data are presented as mean ± SE from biological replicates (*n* = 5 independent experiments). T tillering stage, J jointing stage, R ripening stage.
**Figure S2:** (A) Variation partitioning analysis (VPA) separating the variation of microbiota (prokaryotes, fungi, protists) community structure explained by CCA model. Stage stands for different growth stages, niche represents rhizosphere soils and roots and stress denotes the levels of drought stress. (B) PERMANOVA analysis indicated drought stress treatments and growth stages as factors impacting root‐associated microbiota community structure. *P* values for PERMANOVA were obtained by permutation tests (adonis2; 999 permutations).
**Figure S3:** (A) Prokaryotic co‐occurrence networks in the root under control (CK), moderate drought (MD) and severe drought (SD) treatments. (B) The number or percentage of positive and negative edges between prokaryotes in the root network under control (CK), moderate drought (MD) and severe drought (SD) treatments. (C) The node degree of prokaryotes in the root network under control (CK), moderate drought (MD) and severe drought (SD) treatments. (D) The value of nearest ‐taxon‐index (NTI) of prokaryotes in the root under control (CK), moderate drought (MD) and severe drought (SD) treatments. Different letters indicate significant differences among treatments based on Dunn's post hoc test (BH–FDR adjusted) following the Kruskal–Wallis test.
**Figure S4:** Pie charts showing the 10 zOTUs representing specialists under severe drought stress.
**Figure S5:** Relative abundance of protistan phylum in the bulk soil and rhizosphere soil across different growth stages under control (CK), moderate drought (MD) and severe drought (SD) treatments. TCK control at tillering stage, TMD moderate drought at tillering stage, TSD severe drought at tillering stage, JCK control at jointing stage, JMD moderate drought at jointing stage, JSD severe drought at jointing stage, RCK control at ripening stage, RMD moderate drought at ripening stage, RSD severe drought at ripening stage.

**Table S1:** emi70307‐sup‐0002‐TableS1.xls.

**Table S2:** Relative abundance of dominant prokaryotic zOTUs under control (CK), moderate drought (MD) and severe drought (SD) treatments across different growth stages.

**Table S3:** The correlations of phylum abundances and water holding capacity.

## Data Availability

All raw sequencing data have been deposited to National Center for Biotechnology Information database (NCBI) Sequence Read Archive (SRA) database under the accession numbers PRJNA1189882 (16S), PRJNA1190235 (ITS), and PRJNA1190612 (18S).
