# Supplementary material for: Core Microbial Taxa Strengthen Root Microbial Network Stability Under Drought Stress
Source: Environ Microbiol. 2026 Apr 29;28:e70307. doi: 10.1111/1462-2920.70307 (PMC13128541; doi:10.1111/1462-2920.70307)
Supplement: Supplementary file 1 — Figure S1: (A, B) A schematic diagram of experiment process. The percentage of soil water holding capacity (WHC) under control (CK), moderate drought (MD) and severe drought (SD) treatments. The drought stress treatments began from the second week. The soils and plants were harvested in the tillering stage, jointing stage and ripening stage (the black arrows). (C) The changes in the alpha diversity of prokaryotes, fungi and protists across the bulk soil, rhizosphere soil and root at three different growth stages under control (CK), moderate drought (MD) and severe drought (SD) treatments. Data are presented as mean ± SE from biological replicates (n = 5 independent experiments). T tillering stage, J jointing stage, R ripening stage. Figure S2: (A) Variation partitioning analysis (VPA) separating the variation of microbiota (prokaryotes, fungi, protists) community structure explained by CCA model. Stage stands for different growth stages, niche represents rhizosphere soils and roots and stress denotes the levels of drought stress. (B) PERMANOVA analysis indicated drought stress treatments and growth stages as factors impacting root‐associated microbiota community structure. P values for PERMANOVA were obtained by permutation tests (adonis2; 999 permutations). Figure S3: (A) Prokaryotic co‐occurrence networks in the root under control (CK), moderate drought (MD) and severe drought (SD) treatments. (B) The number or percentage of positive and negative edges between prokaryotes in the root network under control (CK), moderate drought (MD) and severe drought (SD) treatments. (C) The node degree of prokaryotes in the root network under control (CK), moderate drought (MD) and severe drought (SD) treatments. (D) The value of nearest ‐taxon‐index (NTI) of prokaryotes in the root under control (CK), moderate drought (MD) and severe drought (SD) treatments. Different letters indicate significant differences among treatments based on Dunn's post hoc test (BH–FDR adjusted) follo [file EMI-28-e70307-s002.docx]

**Supplementary Material**

**Title**

Core microbial taxa strengthen root microbial network stability under drought stress

**Running title**

Core taxa enhance root microbial network stability

**Authors**

Keren Wu^1,2^, Hang-Wei Hu^1,2^, Dorin Gupta^1^, Yuan Li^3^, Zi-Yang He^1,2^, Feng Wang^1^, Ji-Zheng He^1,2^*

**The affiliation(s) and address(es) of the author(s)**

^1^School of Agriculture, Food and Ecosystem Sciences, Faculty of Science, The University of Melbourne, Parkville, Victoria 3010, Australia

^2^ARC Research Hub for Smart Fertilisers, The University of Melbourne, Parkville, Victoria 3010, Australia

^3^Grasslands and Sustainable Farming, Production Systems Unit, Natural Resources Institute Finland, Halolantie 31A, Kuopio FI−71750, Finland

**Corresponding author**

Dr. Ji-Zheng He

School of Agriculture, Food and Ecosystem Sciences, Faculty of Science, The University of Melbourne, Parkville, Victoria 3010, Australia

ARC Research Hub for Smart Fertilisers, The University of Melbourne, Parkville, Victoria 3010, Australia

Tel: +61390358890

E-mail: [jizheng.he@unimelb.edu.au](mailto:jizheng.he@unimelb.edu.au)

**Figure S1 (A, B)** A schematic diagram of experiment process. The percentage of soil water holding capacity (WHC) under control (CK), moderate drought (MD) and severe drought (SD) treatments. The drought stress treatments began from the second week. The soils and plants were harvested in the tillering stage, jointing stage and ripening stage (the black arrows). **(C)** The changes in the alpha diversity of prokaryotes, fungi, and protists across the bulk soil, rhizosphere soil, and root at three different growth stages under control (CK), moderate drought (MD) and severe drought (SD) treatments. Data are presented as mean ± SE from biological replicates (n = 5 independent experiments). T tillering stage, J jointing stage, R ripening stage.

**Figure S2 (A)** Variation partitioning analysis (VPA) separating the variation of microbiota (prokaryotes, fungi, protists) community structure explained by CCA model. Stage stands for different growth stages, niche represents rhizosphere soils and roots, and stress denotes the levels of drought stress. **(B)** PERMANOVA analysis indicated drought stress treatments and growth stages as factors impacting root-associated microbiota community structure. *P* values for PERMANOVA were obtained by permutation tests (adonis2; 999 permutations).

**Figure S3 (A)** Prokaryotic co-occurrence networks in the root under control (CK), moderate drought (MD) and severe drought (SD) treatments. **(B)** The number or percentage of positive and negative edges between prokaryotes in the root network under control (CK), moderate drought (MD) and severe drought (SD) treatments. **(C)** The node degree of prokaryotes in the root network under control (CK), moderate drought (MD) and severe drought (SD) treatments. **(D)** The value of nearest -taxon-index (NTI) of prokaryotes in the root under control (CK), moderate drought (MD) and severe drought (SD) treatments. Different letters indicate significant differences among treatments based on Dunn’s post-hoc test (BH–FDR adjusted) following the Kruskal–Wallis test.

**Figure S4** Pie charts showing the 10 zOTUs representing specialists under severe drought stress.

**Figure S5** Relative abundance of protistan phylum in the bulk soil and rhizosphere soil across different growth stages under control (CK), moderate drought (MD) and severe drought (SD) treatments. TCK control at tillering stage, TMD moderate drought at tillering stage, TSD severe drought at tillering stage, JCK control at jointing stage, JMD moderate drought at jointing stage, JSD severe drought at jointing stage, RCK control at ripening stage, RMD moderate drought at ripening stage, RSD severe drought at ripening stage.

**
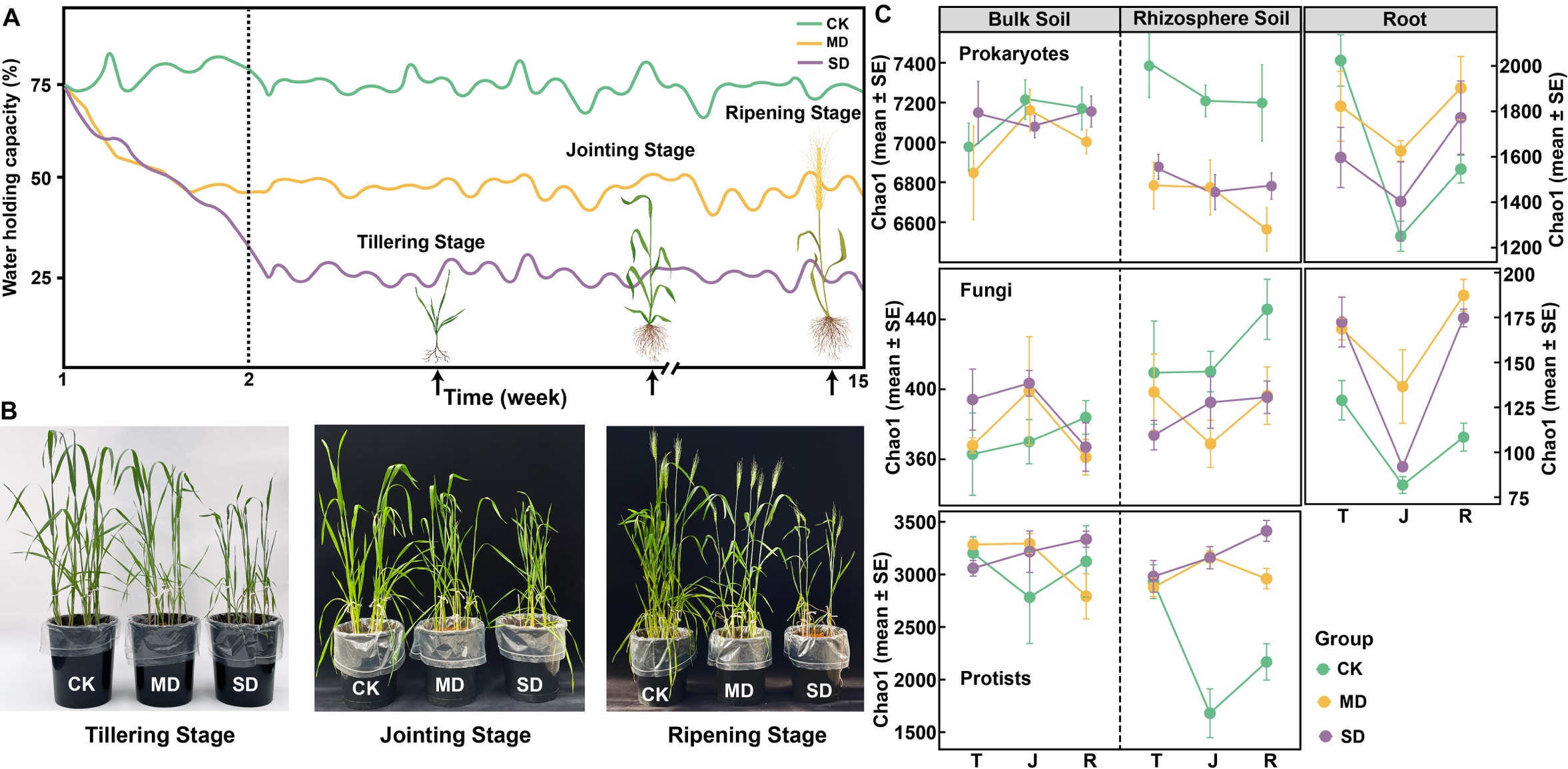
**

**Figure S1**

**
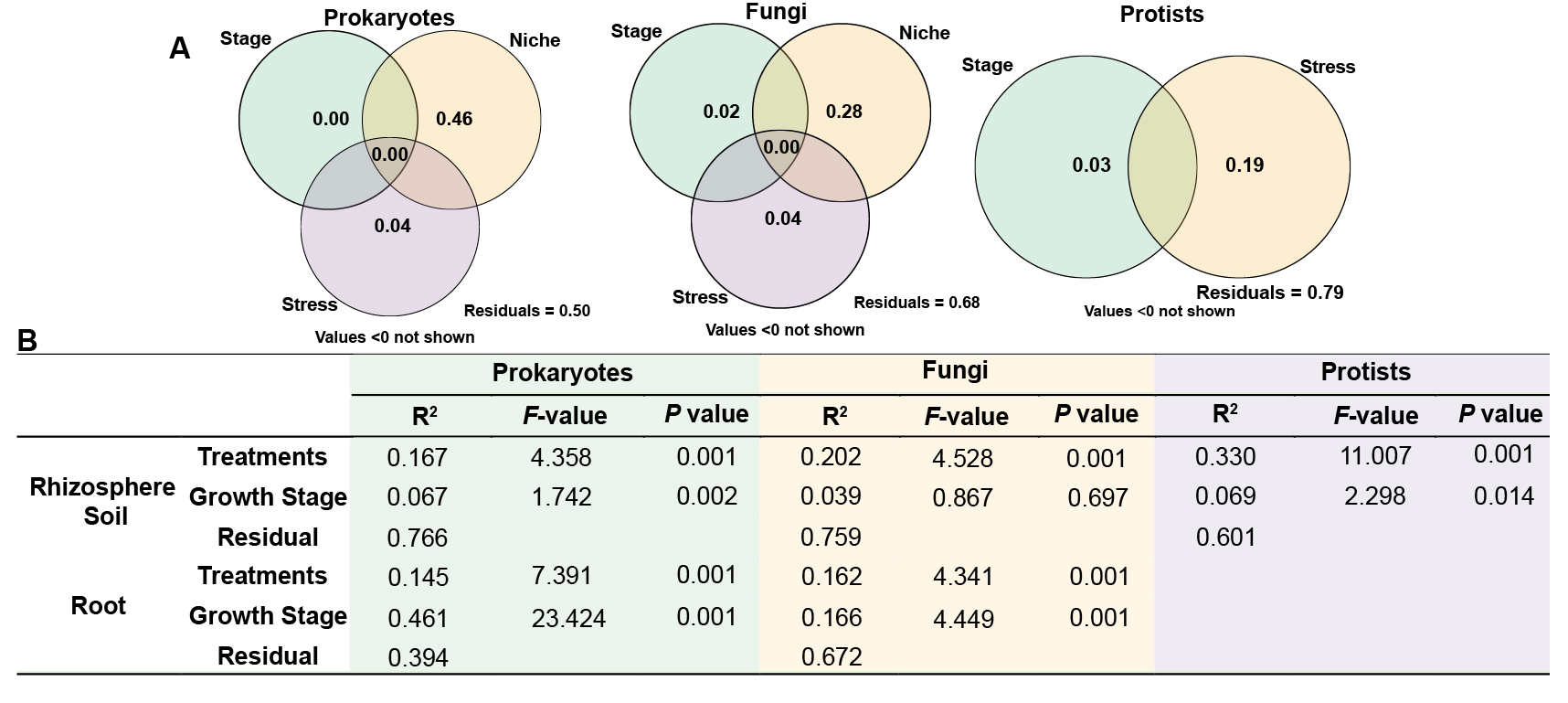
**

**Figure S2**

**
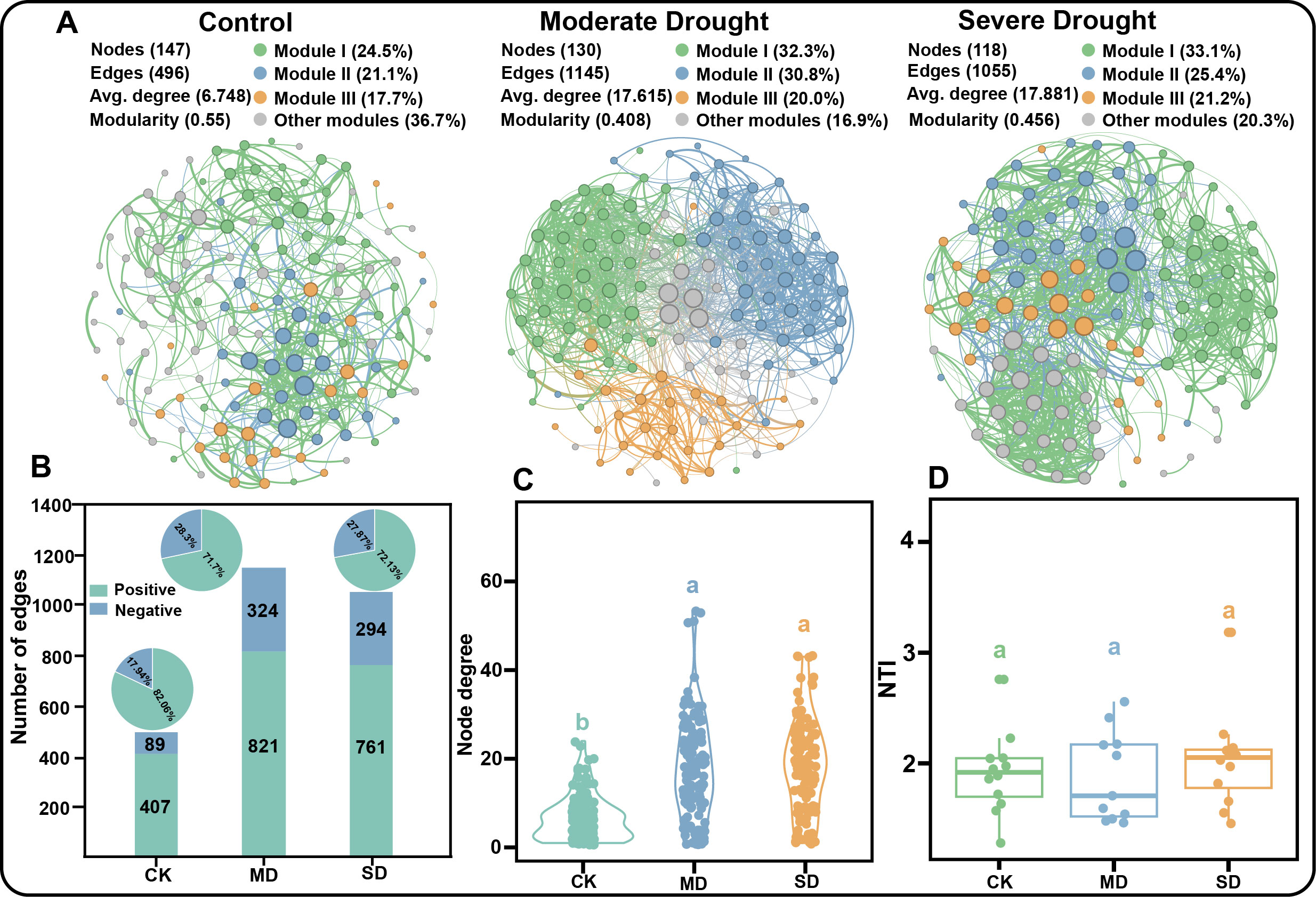
**

**Figure S3**

**
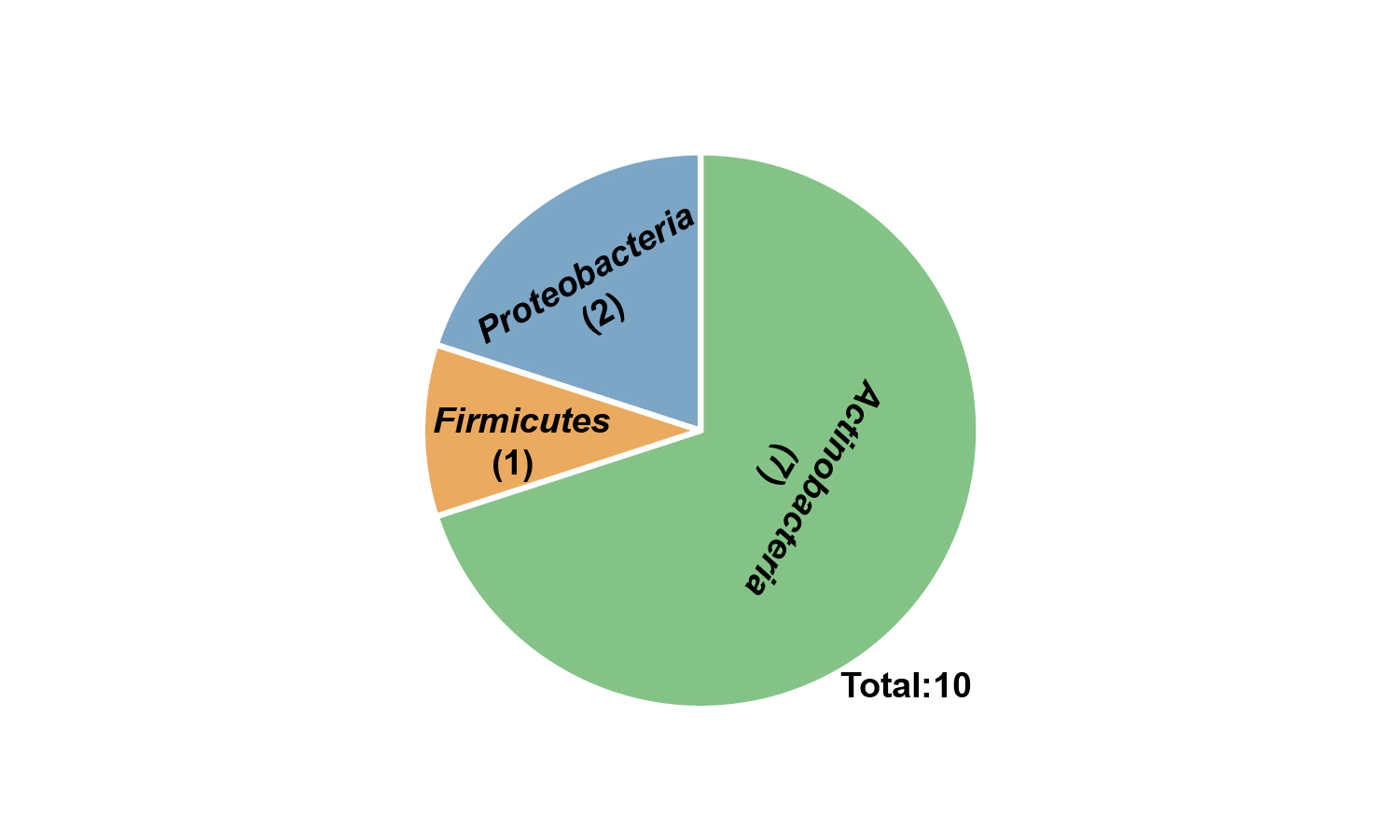
**

**Figure S4**

**
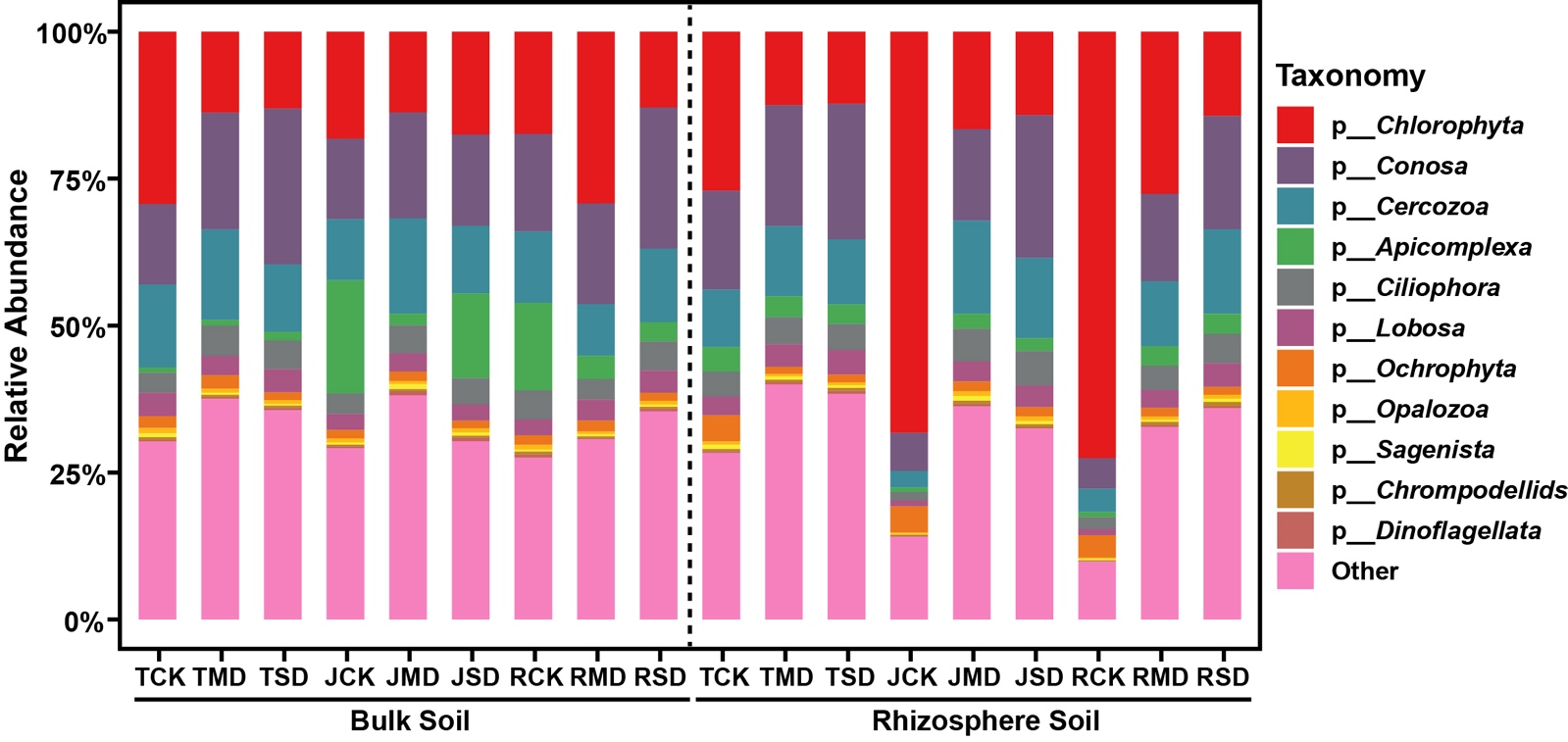
**

**Figure S5**
